# 1st Global Consensus for Clinical Guidelines for the Rehabilitation of the Edentulous Maxilla: Single‐Round Survey on Implant‐Supported Fixed and Removable Prostheses

**DOI:** 10.1111/clr.70027

**Published:** 2026-02-24

**Authors:** Guo‐Hao Lin, Franz J. Strauss, Giulia Brunello, Charlotte Stilwell, Ronald E. Jung, Ina Kopp, Frank Schwarz, Hom‐Lay Wang

**Affiliations:** ^1^ Department of Orofacial Sciences, School of Dentistry University of California San Francisco San Francisco California USA; ^2^ Department of Periodontics & Oral Medicine, School of Dentistry University of Michigan Ann Arbor Michigan USA; ^3^ Clinic of Reconstructive Dentistry, Center of Dental Medicine University of Zurich Zurich Switzerland; ^4^ Faculty of Dentistry, Center for Studies and Innovation in Dentistry Universidad Finis Terrae Santiago Chile; ^5^ Department of Oral Surgery University Hospital of Düsseldorf Düsseldorf Germany; ^6^ Charité – Universitätsmedizin Berlin, Corporate Member of Freie Universität Berlin and Humboldt‐Universität Zu Berlin Department of Orthodontics and Dentofacial Orthopaedics Berlin Germany; ^7^ Division of Gerodontology and Removable Prosthodontics, University Clinics of Dental Medicine University of Geneva Geneva Switzerland; ^8^ AWMF‐Institut für Medizinisches Wissensmanagement Philipps‐Universität Marburg Marburg Germany; ^9^ Department of Oral Surgery, Implantology and Oral Medicine Goethe University Frankfurt am Main Germany

**Keywords:** complete denture, dental implants, implant‐supported dentures, survey

## Abstract

**Objectives:**

This single‐round survey on prosthodontic rehabilitation of the edentulous maxilla is one of four clinician surveys commissioned prior to the first Global Consensus for Clinical Guidelines (GCCG). Within the GCCG aim of refining treatment protocols and enhancing patient care, this survey gathered expert insights into best practices, challenges, and treatment outcomes for these complex restorative procedures.

**Materials and Methods:**

The 25‐item survey was sent to 230 identified experts from 41 countries in October 2024. Participation in the survey was voluntary and without incentives. Informed consent was obtained at the beginning of the survey. All data were collected, stored, and processed anonymously.

**Results:**

Of the 230 experts contacted, 123 accessed the survey and 121 provided consent and completed the questionnaire, resulting in a response rate of 52.6%. The survey responses identified 13 statements that reached consensus (> 75% but ≤ 95% agreement) and five that reached strong consensus (> 95% agreement). These 18 statements were derived from 6 items addressing the topics of anatomical landmarks for tooth positioning, preferred distal implant position, preferred minimum implant diameter, preferred retention method, relevant patient‐reported outcomes, and relevant clinician‐reported outcomes.

**Conclusion:**

This study gathered valuable expert insights to inform a consensus development process for establishing clinical practice guidelines for management of the edentulous maxilla. The survey identified 18 statements from six items that reached consensus or strong consensus among survey respondents. The items not achieving consensus served to reflect real‐world implant treatment strategies, identify literature gaps, and highlight any inconsistencies between expert consensus and current evidence.

## 1 Introduction

With rising life expectancy and population growth, an increasing number of elderly patients are seeking dental care, requiring careful consideration of medical, social, and dental factors for appropriate treatment (Collaborators et al. 2020; Zembic and Wismeijer 2014). Worldwide, a considerable number of edentulous individuals, especially the elderly, are in need of rehabilitation (Curtis et al. 2021).

Interestingly, many studies evaluating clinical outcomes for edentulous patients primarily focus on determining the optimal number of implants needed for implant‐supported removable prostheses (hereafter referred to as implant‐supported overdentures, IODs) (Di Francesco et al. 2019; Lee et al. 2012) or fixed full‐arch prostheses (Daudt Polido et al. 2018; Heydecke et al. 2012). Additionally, there is a greater research emphasis on the fully edentulous mandible compared to the maxilla, likely because mandibular dentures generally have poorer retention and stability (Limpuangthip et al. 2019). Given the limited available evidence, there is an urgent need for a patient‐centered consensus meeting to specifically address the rehabilitation of maxillary full‐arch edentulism. Such a meeting would unite leading experts to develop comprehensive and ideally evidence‐based guidelines that prioritize the specific needs and experiences of patients undergoing maxillary rehabilitation, ultimately enhancing care quality and patient satisfaction (Diseases and Injuries 2024).

In 2025, the first Global Consensus for Clinical Guidelines (GCCG) was established to address edentulism in the maxilla and its impact on oral health and quality of life. This initiative, named “Patient‐Centered Clinical Workflow in Implant Dentistry,” aimed to unite leading global experts to develop evidence‐based, patient‐centered guidelines for rehabilitating the edentulous maxilla. As part of the GCCG project, a single‐round survey method was conducted. Four surveys were developed to gather expert opinions on various aspects of full‐arch maxillary rehabilitation. The fourth survey specifically focused on the restorative rehabilitation of the edentulous maxilla, addressing implant‐supported fixed prostheses and IODs. The goal was to gather expert insights on best practices, challenges, and treatment outcomes for these complex procedures, with the ultimate aim of refining treatment protocols and enhancing patient care.

## 2 Materials and Methods

The current survey employed a single‐round survey method to capture diverse perspectives and interpretations on a specific topic while mitigating negative aspects of group discussions, such as dominant individuals and opinions that can compromise balanced decision‐making (Barrett and Heale 2020). A single‐round survey format was adopted to improve brevity and efficiency.

The Ethical Committee at the University of Düsseldorf approved this single‐round survey study (Protocol no. 2024‐2973). The study followed the guidelines set forth in “Good practice in the conduct and reporting of survey research” (Kelley et al. 2003).

### 2.1 Study Design

This research employed a single‐round survey method conducted in preparation for the GCCG Workshop, which was held in Boston in June 2025. The main goal was to examine emerging trends and developments in the rehabilitation of the edentulous maxilla to align clinical practices with current scientific evidence. By gathering expert opinions, the study sought to reflect real‐world implant treatment strategies, identify literature gaps, and highlight any inconsistencies between expert consensus and current evidence.

The findings from the single‐round survey were used to formulate key questions for future systematic reviews and to guide discussions at the GCCG Workshop in Boston. The Scientific Chairs of the GCCG (Frank Schwarz, Hom‐Lay Wang), together with the Survey Panel (Giulia Brunello, Todd Schoenbaum) and the Cross‐disciplinary Expert and Patient Panel (Franz‐Josef Strauss, Guo‐Hao Lin), were responsible for developing the questionnaire and overseeing the selection and invitation of experts.

### 2.2 Questionnaire

Items listing in the questionnaire for the experts were focusing on fixed implant‐supported prostheses and IODs for the rehabilitation of the edentulous maxilla. The formulation of the questions was based on the topics addressed in the latest literature (Kappel et al. 2021; Kashbour et al. 2015; Lan et al. 2025; Sanz et al. 2023) and therefore a representative rather than a comprehensive reflection of restorative treatment considerations for the patient workflow. The initial questionnaire draft was prepared by the Survey Panel.

To ensure clarity and relevance, the questionnaire underwent an iterative validation process involving multiple rounds of feedback and revision. During its development, the Scientific Chairs, the Survey Panel, and the Cross‐disciplinary Expert and Patient Panel engaged in a series of discussions conducted via online meetings and email correspondence. This collaborative exchange continued until consensus was achieved, and the final version of the questionnaire was approved by all members of the leadership team. Besides the Scientific Chairs, key contributors consisted of the Survey Panel and the Cross‐disciplinary Expert and Patient Panel. Their insights were instrumental in refining the questionnaire prior to receiving ethical approval.

The final questionnaire, written in English and designed to be completed in 15–20 min, consisted of 25 items (see Appendix S1) organized as follows:

#### 2.2.1 Consent and Professional Background

- Declaration of consent (item 1);
- Professional specialization and working environment (items 2–3);

#### 2.2.2 Patient Selection

c Use of IODs (item 9);

#### 2.2.3 Diagnostic Tools

d Anatomical landmark for tooth positioning(s) (item 16);

#### 2.2.4 Treatment Planning

e Preferred implant type (item 5);
f Smallest implant diameter (item 23);
g Most distal implant site for maxillary fixed prostheses (item 15);
h Preferred retention method (item 8);
i Utilization of an angled screw channel system (item 21);
j Timing for delivery of the provisional prosthesis (item 17);
k Utilization of a conventional removable denture for conversion (item 18);
l Fabrication of a single prosthesis or segmented prostheses (item 19);
m Preferred prosthesis material/design in the maxilla (item 7);
n Utilization of milled titanium framework (item 12);
o Utilization of titanium abutments or bases in the prostheses (item 11);
p Utilization of multi‐unit abutments (item 4);

#### 2.2.5 Treatment Procedures

q Impression or scan method (item 13);
r Maximum number of implants when using intraoral scanner (item 14);

#### 2.2.6 Maintenance Care

s Home hygiene instruments/regimens (item 20);
t Interval of removing the definitive prosthesis for evaluation, hygiene, or screw replacement (item 6);
u Frequency of re‐torquing the prosthetic screws (item 10);
v Most encountered or frequent complication (item 22);

#### 2.2.7 Fundamental Outcomes to Be Included in Future Studies

w Relevant patient‐reported outcome measures (PROMs) (item 24);
x Relevant clinician‐reported outcomes (ClinROs) (item 25).

### 2.3 Sample Size, Expert Selection and Data Collection

**FIGURE 1:**
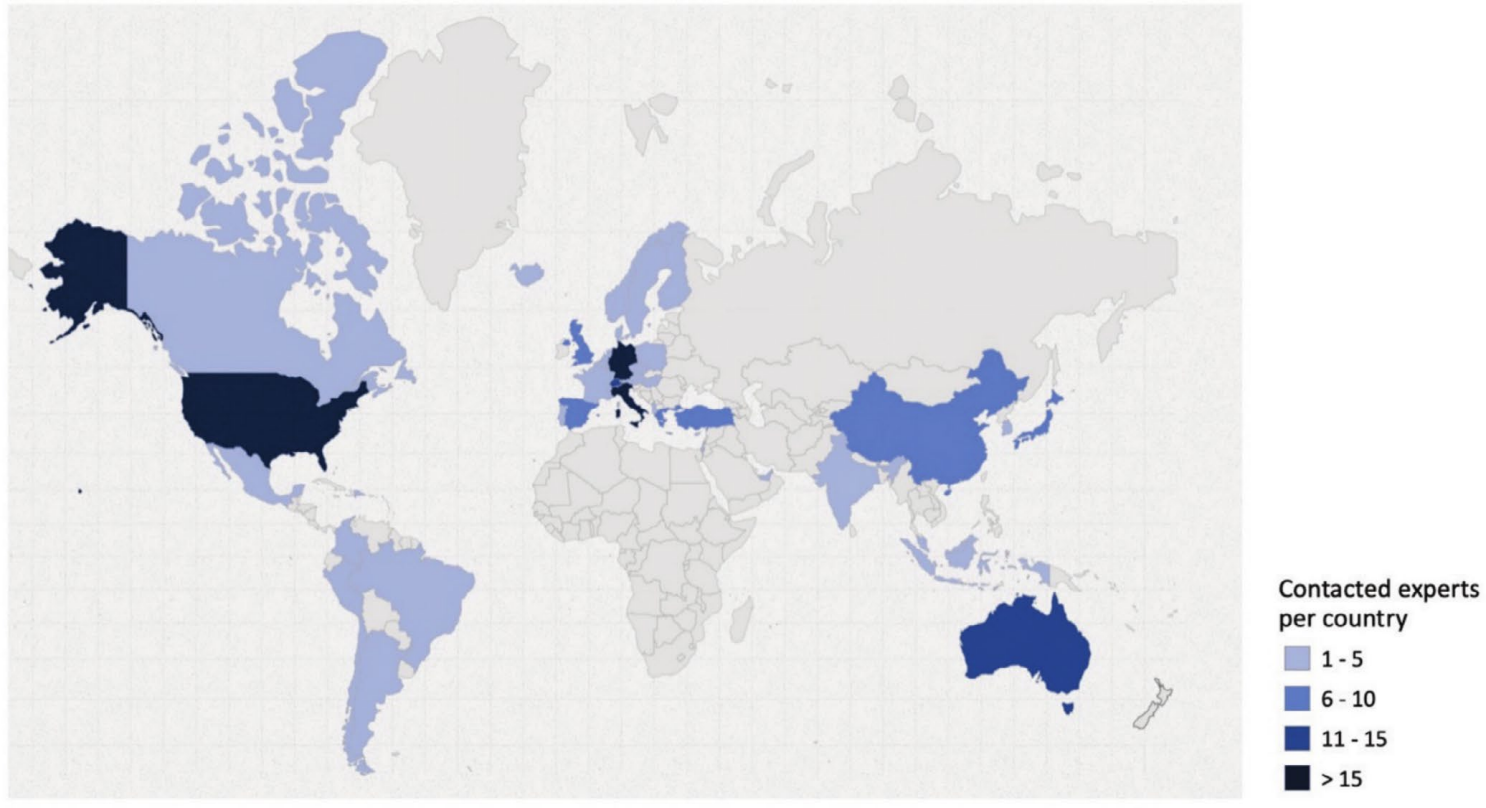
Distribution by country of the contacted experts.

There is no definitive method for determining the number of participants required for a single‐round survey study. Expectations are therefore guided by the COMET Initiative guidelines (Williamson et al. 2017) and previous literature (Alarcon et al. 2021; Madianos et al. 2016; Sanz et al. 2019). Based on an expected response rate of 50%, a total of 230 experts from 41 different countries were identified and contacted for the study. The detailed distribution of these experts by country is shown in Table 1 and Figure 1. Due to the anonymous nature of the data collection, respondents could not be distinguished from non‐respondents. Additionally, information on gender and age was not collected at the time of survey submission and therefore cannot be reported for the invited experts. The survey link was disseminated via email by the European Association for Osseointegration (EAO) Office to experts selected and approved by the Scientific Task Force. These experts were chosen from members or past presenters of leading international implant dentistry organizations, including the Academy of Osseointegration (AO), the EAO, the International Team for Implantology (ITI), and the Osteology Foundation (OF).

**TABLE 1:** Details and distribution by country of the contacted experts.

Country	No.	%
Albania	1	0.4
Argentina	5	2.3
Australia	14	6.1
Austria	1	0.4
Belgium	5	2.3
Brazil	5	2.3
Canada	3	1.3
Chile	3	1.3
China	8	3.5
Colombia	2	0.9
Cyprus	1	0.4
Czech Republic	1	0.4
Denmark	2	0.9
Dominican Republic	1	0.4
Finland	1	0.4
France	3	1.3
Germany	17	7.4
Greece	6	2.6
Hungary	1	0.4
Iceland	1	0.4
India	2	0.9
Indonesia	1	0.4
Israel	1	0.4
Italy	17	7.4
Japan	8	3.5
Republic of Korea	2	0.9
Luxembourg	1	0.4
Malaysia	1	0.4
Mexico	3	1.3
Netherlands	3	1.3
Norway	3	1.3
Peru	1	0.4
Poland	3	1.3
Portugal	4	1.7
Spain	10	4.3
Sweden	1	0.4
Switzerland	15	6.5
Türkiye	6	2.6
United Arab Emirates	1	0.4
United Kingdom	9	4.0
United States of America	57	24.8

The single‐round survey began on October 3, 2024, and remained open until October 25, 2024, spanning 3 weeks. A reminder was sent on October 19, 2024. Participation in the survey was voluntary and without incentives. The survey was conducted electronically using Microsoft FORMS (Redmond, WA, USA). Informed consent was obtained from all respondents at the beginning of the survey; if consent was not given, the survey was automatically terminated. All data were collected, stored, and processed anonymously.

### 2.4 Agreement Definition

Although there is no universally accepted method for defining consensus in survey studies (von der Gracht 2012), this study adopted the following thresholds for items using a 7‐point Likert scale:

− *strong consensus*: achieved when > 95% of the experts “somewhat agreed”, “agreed” or “strongly agreed” with the statement made, or alternatively when > 95% “somewhat disagreed”, “disagreed” and “strongly disagreed”;
− *consensus*: defined when either agreement or disagreement was greater than 75% and no more than 95%;
− *no consensus*: defined when either agreement or disagreement was 75% or less.

Similar for multiple‐choice items, allowing the selection of either one or multiple applicable statements (items 9, 16, 20, 24, and 25), strong consensus for any single statement was defined as selection by more than 95% of respondents; consensus as selection by more than 75% and no more than 95%; and no consensus as 75% or less (Diamond et al. 2014).

### 2.5 Statistical Analysis

Each survey item was analyzed independently using descriptive statistics, with results expressed as percentages. Additionally, the level of agreement for each item was reported using the median score and interquartile range (IQR), in accordance with the RAND guidelines (Khodyakov et al. 2023).

In the graphs, the percentage of agreement (%) was determined by combining the responses “somewhat agree,” “agree,” and “strongly agree.” If the total of “somewhat disagree,” “disagree,” and “strongly disagree” was higher, that percentage was reported instead, with clarifications provided in the Figure footnotes.

Data analysis was performed using a computer software (Stata version 18, StataCorp LLC, College Station, Texas, USA).

## 3 Results

Out of 230 experts contacted, 123 accessed the survey. Of these, 121 provided consent and completed the questionnaire, resulting in a response rate of 52.6%. Although efforts were made to ensure broad geographical representation among participants, the proportion of participating experts from North America, South America, and Europe was higher than that of those from Asia and Africa (Figure 1).

### 3.1 Consent and Professional Background

The majority of the respondents reported having either one (71.9%) or multiple (21.5%) specialization degrees, whereas only eight respondents (6.6%) identified themselves as general practitioners. The most frequently reported specialty was Prosthodontics (67.8%), followed by Periodontology (24.8%), Oral Surgery (19.8%), and Oral & Maxillofacial Surgery (4.1%). Regarding the working environment, the most commonly selected option was “university” (70.2%), followed by “private clinic” (66.9%), “public hospital” (9.9%), and “other” (1.7%). Approximately half of the experts (52.1%) worked in only one setting: 32 at university, 30 in private clinics, and 1 in a public hospital.

### 3.2 Patient Selection

#### 3.2.1 Use of IODs

For the edentulous maxilla, 64.5% of respondents used IODs (locators/bar attachments) when alveolar volume was insufficient for lip support; 16.5% preferred them routinely, 15.7% never used them, and 3.3% chose other. Notably, 33.1% cited financial considerations as a factor.

### 3.3 Diagnostic Tools

#### 3.3.1 Anatomical Landmark for Tooth Positioning(s)

Facial midline and upper lip were the most used landmarks (94.2%), followed by lips (67.8%) and eyes (66.9%). Nose (32.2%) and retromolar pad (21.5%) were less common. Consensus (94.2%) confirmed the use of the facial midline and upper lip as primary reference points. Although no consensus was reached, the respondents considered both the upper and lower lips as secondary reference points (67.8%).

### 3.4 Treatment Planning

#### 3.4.1 Preferred Implant Type

Regarding implant type preferences for maxillary fixed full‐arch implant‐supported prostheses, 66.1% preferred bone‐level implants, 16.5% preferred tissue‐level implants, 15.7% were ambivalent about the two, and 1.7% selected other.

#### 3.4.2 Smallest Implant Diameter

Assuming a “normal” or healthy occlusal scenario, 6.6% preferred 2.9–3.0 mm, 81.0% 3.5 mm, 7.4% 4 mm, and 5% selected other diameters. None preferred diameters of 2 mm or less. Consensus supported 3.5 mm as the smallest acceptable diameter for maxillary fixed full‐arch implant‐supported prostheses.

#### 3.4.3 Most Distal Implant Site for Maxillary Fixed Prostheses

Assuming sufficient bone is available or can be augmented, 81.8% preferred the first molar as the distal site; consensus was reached in favor of the first molar position.

#### 3.4.4 Preferred Retention Method

For a maxillary fixed full‐arch implant‐supported prosthesis, there was expert consensus (93.4%) for screw‐retained prostheses, while 4.1% preferred cemented prostheses, and 2.5% selected other options.

#### 3.4.5 Utilization of an Angled Screw Channel System

Regarding angled screw channel systems, 12.4% always used them, 59.5% sometimes, 21.5% rarely, and 6.6% never.

#### 3.4.6 Timing for Delivery of the Provisional Prosthesis

In delivering provisional prostheses, 45.5% reported to do it on the same day as implant placement; 15.7% one day after; 17.4% within the first week; and 19.8% after implant osseointegration. Another 1.6% reported other options.

#### 3.4.7 Utilization of a Conventional Removable Denture for Conversion

When asked about converting conventional removable dentures into fixed provisionals, 9.9% reported always doing so; 61.2% sometimes; and 28.9% reported never.

#### 3.4.8 Fabrication of a Single Prosthesis or Segmented Prostheses

For maxillary fixed full‐arch implant‐supported prostheses, 19.0% of respondents always fabricated a single piece, 54.5% mostly, 19.8% occasionally, and 6.7% never did.

#### 3.4.9 Preferred Prosthesis Material/Design in the Maxilla

For maxillary fixed full‐arch implant‐supported prostheses, preferences included full zirconia with titanium bases (39.7%), zirconia with custom titanium framework (31.4%), ceramo‐metal (11.6%), acrylic/metal (9.9%), and full zirconia without titanium bases (4.1%).

#### 3.4.10 Utilization of Milled Titanium Framework

Regarding milled titanium framework, 22.3% reported always using them; 60.3% sometimes, 9.9% never, and 7.5% noted it was not applicable since they did not use zirconia prostheses.

#### 3.4.11 Utilization of Titanium Abutments or Bases in the Prostheses

In terms of using titanium abutments or bases, 66.1% reported always using them; 19.0% sometimes; 4.1% never. An additional 10.8% indicated it was not applicable since they did not prefer to use zirconia prostheses.

#### 3.4.12 Utilization of Multi‐Unit Abutments

For maxillary fixed full‐arch implant‐supported prostheses, 62.0% of the respondents used them on all implants, 24.8% only on angled implants, and 4.1% never.

### 3.5 Treatment Procedures

#### 3.5.1 Impression or Scan Method

For fabricating the master model, 41.3% of the respondents preferred splinted open‐tray copings, 26.4% used intraoral scans, and smaller percentages used other methods.

#### 3.5.2 Maximum Number of Implants When Using Intraoral Scanner

When using intraoral scanners (excluding photogrammetry) to fabricate the master cast for multiple implants planned to be part of the same prosthesis, 40.5% reported no maximum number of implants, 29.8% set a limit at 3 implants per quadrant, 6.6% at 4 implants per arch; another 6.6% at 2 implants per quadrant, and 5.8% at 5 implants per arch as the maximum number. Additionally, 10.7% of the respondents did not use intraoral scanners.

### 3.6 Maintenance Care

#### 3.6.1 Home Hygiene Instruments/Regimens

The most commonly recommended hygiene aids were super floss (72.7%), proxy brushes (72.7%), electric toothbrushes (61.2%), and water flossers (58.7%). Additionally, 47.0% of the respondents recommended a manual toothbrush. A small portion of respondents (3.3%) other hygiene instruments or regimens not listed above.

#### 3.6.2 Interval of Removing the Definitive Prosthesis for Evaluation, Hygiene, or Screw Replacement

When asked about removing the definitive prosthesis for evaluation, hygiene, or screw replacement, 38% of the respondents reported only when signs or symptoms require it; 24.8% every 1–3 years; 19.0% annually; 6.6% every 3–5 years; 5.0% at every visit; and another 5.0% never. The remaining 1.6% reported other intervals.

#### 3.6.3 Frequency of Re‐Torquing the Prosthetic Screws

Regarding re‐torquing prosthetic screws, 44.6% reported almost never doing so; 27.3% did so within 1 month after delivery, 17.4% within 1 year of delivery, and 10.7% reported never re‐torquing.

#### 3.6.4 Most Encountered or Frequent Complication

For a fixed, full‐arch implant‐supported prosthesis, the most frequent complication was fracture of veneering porcelain (58.7%) followed by screw loosening (23.1%), debonding of titanium bases or abutments (9.1%), framework fracture (8.3%), and broken screws (0.8%).

### 3.7 Fundamental Outcomes to be Included in Future Studies

#### 3.7.1 Relevant Patient‐Reported Outcome Measures (PROMs)

**FIGURE 2:**
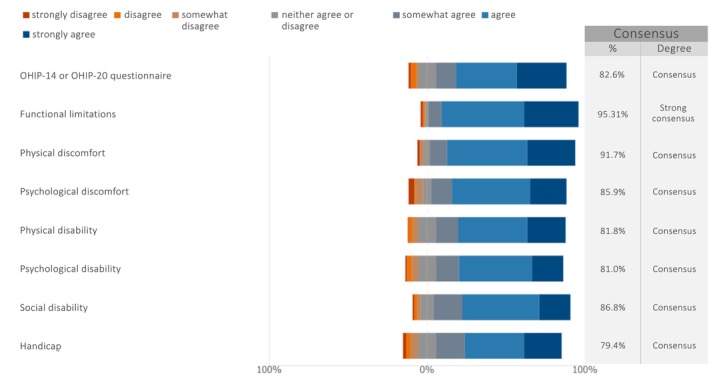
In future studies on maxillary full‐arch rehabilitation with dental implants, how relevant do you consider the following patient‐reported outcome measures (PROMs)?

When asked about the PROMs to be considered for future studies on maxillary full‐arch rehabilitation with dental implants (Figure 2), strong consensus was achieved on functional limitations (median: 6; IQR: 6–7). Consensus was also reached for all other listed PROMs: the OHIP‐14 or OHIP‐20 questionnaire (median: 6; IQR: 5–7), physical discomfort (median: 6; IQR: 6–7), psychological discomfort (median: 6; IQR: 5–6), physical disability (median: 6; IQR: 5–6), psychological disability (median: 6; IQR: 5–6), social disability (median: 6; IQR: 5–6) and handicap (median: 6; IQR: 5–6). A summary graph displaying the median and IQR was created following the RAND guidelines (Khodyakov et al. 2023) and presented in the Figure S1.

#### 3.7.2 Relevant Clinician‐Reported Outcomes (ClinROs)

**FIGURE 3:**
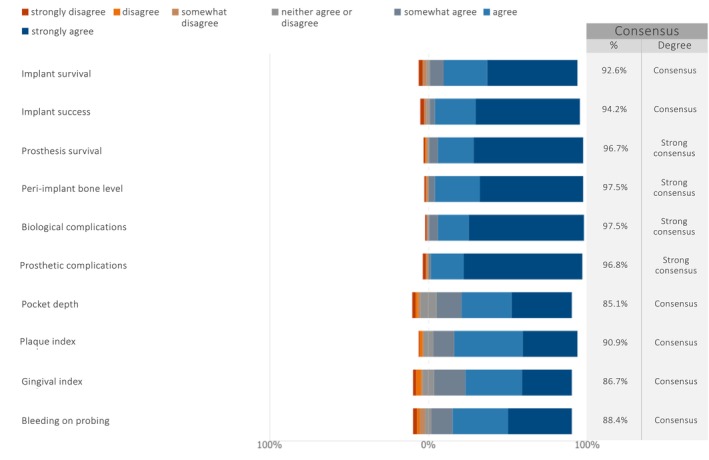
In future studies on maxillary full‐arch rehabilitation with dental implants, how relevant do you consider the following clinician‐reported outcomes (ClinROs)?

When asked about the ClinROs in future studies on maxillary full‐arch rehabilitation with dental implants (Figure 3), strong consensus was achieved on four ClinROs: prosthesis survival (median: 7; IQR: 6–7), peri‐implant bone level (median: 7; IQR: 6–7), biological complications (median: 7; IQR: 6–7), and prosthetic complications (median: 7; IQR: 6–7). Consensus was also achieved on all other listed ClinROs: implant survival (median: 7; IQR: 6–7), implant success (median: 7; IQR: 6–7), pocket depth (median: 7; IQR: 5–7), plaque index (median: 7; IQR: 6–7), gingival index (median: 7; IQR: 5–7), and bleeding on probing (median: 7; IQR: 5.5–7). Summary graphs displaying the median and IQR were created following the RAND guidelines (Khodyakov et al. 2023) and are presented in the Figure S2.

A summary of the results for all items is provided in Appendix S2.

## 4 Discussion

### 4.1 Study Design

This research utilized a single‐round survey method to prepare for the GCCG Workshop in Boston, June 2025. This single‐round survey was focused on obtaining responses and perspectives from respondents actively engaged in the rehabilitation of the edentulous maxilla. Whilst the response rate was encouraging (52.6%), it should be acknowledged that the total number of respondents was limited to 121; the results should thus be interpreted accordingly.

### 4.2 Questionnaire Design

The questionnaire for the respondents focused on implant‐supported prostheses and IODs for edentulous maxilla rehabilitation. The 22 clinically focused survey items (items 4–25) must be considered to be representative rather than a comprehensive examination of current treatment planning and clinical decision‐making. At the same time, as the formulation of these survey items was based on restorative treatment considerations raised in recent literature, the survey responses also served to highlight alignment and discrepancies between expert opinion and the literature, together with identifying gaps in the latter to be explored further. Consequently, a number of research questions were proposed for future investigations (see Section 4.7).

### 4.3 Survey Participation

With the aim of examining global trends and developments in edentulous maxilla rehabilitation, the 230 invited experts were deliberately selected to represent a wide geographical spread of responders across 41 countries. With the anonymous response, however, it is not possible to confirm the extent of the actual global representation achieved.

### 4.4 Professional Background

The identification of the experts from the 41 different countries contacted for the study was based on their membership or past contribution as presenters of four leading international implant dentistry organizations (AO, EAO, ITI and OF). Although recognizing that this may have introduced some selection bias, concerted efforts were also made to ensure as balanced a representation as possible amongst the contacted experts in terms of specialties and regions.

### 4.5 Items Reaching Consensus

#### 4.5.1 Diagnostic Tools

##### 4.5.1.1 Anatomical Landmark for Tooth Positioning(s)

There was a consensus that the facial midline and upper lip in combination serve as the primary anatomical landmarks positioning teeth in maxillary fixed full‐arch implant‐supported prostheses. This finding partially aligns with a previous study (Bidra et al. 2009) that identified the midline of the oral commissures and the upper lip (tip of the philtrum) as reliable landmarks near the facial midline during smiling. Similarly, another study (Farahani et al. 2019) confirmed that the oral commissures showed the least deviation from the facial midline, surpassing landmarks such as the nasion and philtrum. Although the midline of the oral commissures was not included as a response in the distributed questionnaire for analyzing anatomical references for prosthesis due to their variability and potential for dynamic movement, future investigations should assess its consistency and accurate alignment. Additionally, positioning the anterior tooth midline in alignment with the facial midline is recommended, as deviations between these midlines are more easily detected (Cardash et al. 2003).

#### 4.5.2 Treatment Planning

Among the 14 items related to treatment planning, only three achieved consensus: (1) the minimum implant diameter should be 3.5 mm; (2) the most distal implant site for maxillary fixed prostheses should be the first molar; and (3) the preferred retention method should be screw‐retained. A detailed discussion of each of these consensus items is provided in the following sections.

##### 4.5.2.1 Smallest Implant Diameter

The respondents reached a consensus that a 3.5 mm implant is the minimum acceptable implant diameter for supporting maxillary fixed full‐arch implant‐supported prostheses in normal occlusal conditions. Although this treatment approach has been widely used to restore both function and esthetics, there is limited literature on the ideal implant diameter. Early case series (Crespi et al. 2012; Malo et al. 2012, 2005) reported 5‐year implant survival rates exceeding 90% for implants between 3.3 and 4.0 mm. However, two systematic reviews (Schiegnitz and Al‐Nawas 2018; Storelli et al. 2021) have found no significant difference in the 5‐year survival rate between narrow‐diameter (< 3.5 mm) and standard‐diameter implants when supporting fixed full‐arch maxillary prostheses. Given the insufficient long‐term evidence for narrow‐diameter implants (Schiegnitz and Al‐Nawas 2018) and methodological limitations in available studies, clinicians should be cautious when considering narrow‐diameter implants.

##### 4.5.2.2 Most Distal Implant Site for Maxillary Fixed Prostheses

There was a consensus that the preferred position for the most distal implant for a fixed full‐arch maxillary prosthesis is the first molar region. Clinical evidence supporting this, however, is limited. A recent cohort study (Sakata et al. 2025) compared implant placement in the first and second molar regions in patients with Kennedy Class II dentition and concluded that implants up to the first molar might be sufficient. Improvement in bite force, masticatory function, oral health‐related quality of life (OHRQoL), and nutritional intake were similar between the groups. Another 5‐year randomized controlled trial also recommended first molar occlusion for optimal biomechanical advantages (Toia et al. 2025). Conversely, a study (Nam et al. 2014) reported increased masticatory ability and satisfaction with implant restoration extending to the second molar in partially edentulous patients. Although most respondents (81.8%) favored placement at the first molar region, these mixed findings indicate the need for future clinical trials to further validate this recommendation.

##### 4.5.2.3 Preferred Retention Method

There was a consensus among the respondents that screw‐retained prostheses are the preferred retention method for maxillary fixed full‐arch implant‐supported prostheses. This consensus likely stems from the retrievability and ease of maintenance associated with screw‐retained prostheses (Bidra et al. 2016; Monje et al. 2016). A recent systematic review (Gaddale et al. 2020) concluded that full‐arch screw‐retained prostheses offer easier retrievability compared to those cemented, thereby allowing for simpler management of technical and biological complications. Similarly, another systematic review found that screw‐retained reconstructions exhibited fewer technical and biological complications overall; although no statistical difference was noted between cemented and screw‐retained reconstructions regarding survival or failure rates (Wittneben et al. 2014). Consequently, these restorations are favored due to their ease of treatment and biological compatibility.

#### 4.5.3 Fundamental Outcomes to be Included in Future Studies

##### 4.5.3.1 Relevant Patient‐Reported Outcome Measures (PROMs)

A strong consensus was reached on the importance of functional limitations as a key PROM for future studies on maxillary full‐arch rehabilitation with dental implants. Additionally, consensus was also achieved on all other proposed PROMs, including the OHIP‐14 or OHIP‐20 questionnaire, physical discomfort, psychological discomfort, physical disability, psychological disability, social disability, and handicap. These findings align with the recent multi‐stakeholder effort aiming to identify a core outcome set and measurements in implant dentistry (ID‐COSM) initiative (Sanz et al. 2023), which utilized the single‐round survey methodology to gather broad input for establishing an internationally recognized core outcome set. This initiative resulted in the identification of 13 essential outcomes categorized into nine domains: function, surgical complications, loss of tissue health, adverse device events, implant/restoration survival and success, implant loss/failure/fracture, quality of life, overall satisfaction, and maintenance effort. These standardized outcome measures will enhance consistency and comparability across studies, ultimately improving the assessment of patient‐centered success in implant dentistry.

##### 4.5.3.2 Relevant Clinician‐Reported Outcomes (ClinROs)

Strong consensus was reached for four key ClinROs: prosthesis survival, peri‐implant bone level, biological complications, and prosthetic complications. Consensus was also achieved on all other listed ClinROs, including implant survival, implant success, pocket depth, plaque index, gingival index, and bleeding on probing. Although not all of these ClinROs were incorporated into the ID‐COSM core set (Sanz et al. 2023), several overlap. It is important to emphasize that while ID‐COSM provides a valuable framework, it should not limit clinical trials exclusively to those outcomes. Researchers are encouraged to tailor outcome selection to their specific hypotheses and objectives, while remaining open to updating core outcomes as new evidence emerges (Powers 3rd et al. 2017; Sanz et al. 2023).

### 4.6 Items Not Reaching Consensus

Several items failed to achieve consensus, reflecting variability in clinical decision‐making for edentulous maxilla rehabilitation. These include the indication for maxillary full‐arch IODs, the preferred implant type, the utilization of an angled screw channel system, the timing for delivery of the provisional prosthesis, the utilization of a conventional removable denture for conversion, the fabrication of a single prosthesis or segmented prostheses, the preferred prosthesis material/design in the maxilla, the utilization of milled titanium framework, the utilization of titanium abutments or bases in the prostheses, the utilization of multi‐unit abutments, impression or scan techniques, the maximum number of implants when using intraoral scanner, home hygiene instruments/regimens, the interval of removing the definitive prosthesis for evaluation, hygiene, or screw replacement, the frequency of re‐torquing the prosthetic screws, and the most encountered or frequent complication.

Majority agreement (> 50% but ≤ 75% agreement) was noted for several items; however, these include selecting an IOD when anterior alveolar volume is insufficient (64.5% of respondents), the use of the upper and lower lips (67.8%) and eyes (66.9%) as anatomical landmarks, occasional use of angled screw channel systems (59.5%) in maxillary definitive full‐arch prostheses, preference for a one‐piece full‐arch prosthesis (54.5%), occasional utilization of milled titanium frameworks (60.3%), consistent use of titanium abutments or bases (66.1%), and use of multi‐unit abutments for maxillary fixed full‐arch implant‐supported prostheses (62.0%). Regarding hygiene recommendations, most respondents endorsed super floss (72.7%), proxy brushes (72.7%), electric toothbrushes (61.2%), and water flossers (58.7%). Lastly, although it did not reach the level of consensus, fracture of veneering porcelain (58.7% of respondents) was reported as the most common complication. These areas highlight opportunities for future research and guideline development.

#### 4.6.1 Patient Selection

In further discussion of the majority agreement rather than actual consensus regarding patient selection for the use of IODs, no formal guidelines exist for their utilization to rehabilitate a fully edentulous maxilla. Several indications, however, have been proposed, and these include: (1) younger patients within the older population who are currently wearing maxillary complete dentures, (2) elderly edentulous patients seeking improved stabilization of their dentures, and (3) patients with acquired or congenital defects (Mericske‐Stern 1998). Long‐term studies (Lo‐Sardo et al. 2025; Sude et al. 2025) have also suggested high implant survival rates and patient satisfaction for these prostheses. However, when comparing IODs to implant‐supported fixed prostheses, a clinical study found that among patients with a similar number of implants, those treated with overdentures reported lower satisfaction and poorer oral health‐related quality of life than those who received fixed prostheses (Brennan et al. 2010). Unlike IODs for the edentulous mandible, however, the specific impact of different retentive options for IODs in patients with an edentulous maxilla (stud attachments, bars, magnetic, or telescopic attachments) on improving patient satisfaction and other ClinROs remains a clinical question yet to be answered.

#### 4.6.2 Treatment Planning

Experts did not reach a consensus on the use of tissue‐level versus bone‐level implants. A retrospective study (Pera et al. 2023) found comparable clinical outcomes after a 12‐month observation period. Nonetheless, a majority agreement favored the use of bone‐level implants for fixed implant‐supported prostheses, possibly due to their ability to provide a more favorable vertical dimension for restorative space. Although data comparing bone‐level and tissue‐level implants remain heterogeneous, a recent review (Lago et al. 2023) suggested that tissue‐level implants may be more suitable for IODs or fixed detachable prostheses.

No consensus or majority agreement was reached on prosthetic material or design for the maxilla. Although zirconia prostheses have been associated with high mechanical strength, reduced plaque accumulation (Chopra et al. 2024; Curiel‐Aguilera et al. 2023) and comparable 10‐year survival rates (Sailer et al. 2018) to metal‐ceramic prostheses, their limitations include a lack of long‐term data, challenges in adhesive cementation, and the need for esthetics optimization (Sadowsky 2020).

There was no consensus related to the use of a single prosthesis versus segmented prostheses. However, a majority of experts favored the use of a single full‐arch prosthesis over segmented designs. Clinical studies directly comparing outcomes between single‐piece and segmented prostheses are scarce. Single‐piece full‐arch prostheses typically require fewer implants; however, increased material thickness is often necessary to prevent fracture, which may compromise comfort and feel less natural during speaking or eating. On the contrary, a retrospective study found that full‐arch bridges are approximately six times more likely to fracture over a 5‐year period compared to 4‐unit bridges (Linkevicius et al. 2008), potentially supporting the rationale for clinicians who favor segmented prostheses.

No consensus was found on titanium abutments or milled titanium frameworks. Nonetheless, a majority of the respondents reported occasionally using milled titanium frameworks and consistently incorporating titanium abutments or bases. However, studies have shown that zirconia abutments with titanium bases perform comparably to metal abutments in terms of survival rates (Bidra et al. 2018; Kim et al. 2013; Sailer et al. 2009). With the advancement of ceramics in dentistry, monolithic zirconia full‐arch prostheses veneered with gingival feldspathic porcelain have demonstrated promising mid‐term outcomes, including high survival rates, fewer mechanical issues, reduced lab costs, improved wear resistance, better fit due to digital workflows, ease of duplication, and less plaque accumulation when compared to resin‐metal prostheses (Chopra et al. 2024; Sadowsky 2020). Further studies are needed to confirm the long‐term performance of zirconia abutments and frameworks and to expand their clinical use.

Angled screw channel systems were occasionally used by 59.5% of respondents, but no trials support their use in full‐arch prostheses. For single implant restorations, two systematic reviews (Cheng et al. 2024; Rasaie et al. 2022) indicated that angled screw channel systems are comparable to straight screw channel systems regarding mechanical and biological complications. However, the long‐term reliability of angled screw channel restorations remains lacking.

Although no consensus was reached, respondents had a majority agreement in using multi‐unit abutments for maxillary fixed full‐arch implant‐supported prostheses. Multi‐unit abutments are used when standard straight or angled abutments do not align with the planned prosthesis, helping to achieve optimal esthetics, a passive fit, and healthy, harmonious peri‐implant tissue contours (Pakhshan and Zahra 2025). Despite their advantages, long‐term performance data remain sparse.

#### 4.6.3 Treatment Procedures

Neither of the two items on impression/scanning reached consensus. Although some studies (Cheng et al. 2024; Pozzi et al. 2024) have suggested that photogrammetry may offer superior precision and accuracy compared to conventional impressions and intraoral scanning, other investigations (Cai et al. 2024; Tallarico et al. 2019) have reported comparable accuracy between conventional impression techniques and intraoral scanning. Notably, no studies to date have specifically evaluated the ideal number of implants when utilizing an intraoral scanner.

#### 4.6.4 Maintenance Care

None of the four maintenance items reached consensus. A variety of devices and materials are available to support home oral hygiene, including manual toothbrushes, counter‐rotational powered brushes, sonic brushes, single‐tuft brushes, interdental brushes, dental floss, toothpaste, oral irrigators, and mouth rinses (Perussolo and Donos 2024). The selection and use of these tools should be customized to each patient's specific needs through proper instruction. Nevertheless, current evidence shows that no standardized protocol exists for oral hygiene practices around dental implants, and it remains inconclusive on the best tools or care frequency in preserving peri‐implant health and reducing the risk of disease recurrence (Herrera et al. 2023).

Regarding prosthesis removal and screw re‐torquing intervals, it has been suggested that patients deemed higher risk due to factors such as age, ability to perform oral self‐care, or biological or mechanical complications with implant‐borne restorations should undergo professional dental examinations more frequently than every 6 months (Bidra et al. 2016). An in vitro study (Varvara et al. 2020) suggested that retightening abutments 2 min after prosthesis delivery significantly reduces preload loss. However, there is limited evidence regarding the optimal timing for removing definitive prostheses for evaluation or the recommended frequency for re‐torquing prosthetic screws.

Common complications associated with implant‐supported prostheses include mechanical and biological issues such as loosening of the overdenture retentive mechanism, implant loss in irradiated maxillae, hemorrhage‐related complications, and resin veneer fractures, among others. Although these have been identified as some of the most frequently encountered complications (Goodacre et al. 2003, 1999), focused studies on full‐arch prosthesis complications remain limited and inconclusive.

### 4.7 Study Limitations and Proposed Additional Research Questions

The study design employs a single‐round survey to capture expert opinions on a complex and multifactorial topic: rehabilitation of the edentulous maxilla, where robust clinical evidence remains scarce. The justification for this approach was two‐fold: (1) seeking to redress the inconsistency in clinical management and the lack of large‐scale trials through the collection of subjective yet informed perspectives to guide future research directions and (2) enhancing response rate and minimizing participant burden. In terms of the perspectives collected, however, it must be acknowledged that the relatively modest sample size of 121 respondents representing just over half of the experts contacted (response rate 52.6%) imposes limitations on the generalizability and statistical strength of the findings. Additionally, the adoption of a single‐round survey methodology may have limited the opportunity for iterative consensus development typical of traditional Delphi methods (Humphrey‐Murto and de Wit 2019; Quirke et al. 2023).

Although efforts were made to ensure broad geographical representation among the contacted experts, it must be acknowledged that a higher proportion of invitees were from North America, South America, and Europe compared to Asia and Africa. Additionally, because the survey responses were anonymous, it was not possible to analyze the actual geographical distribution of the 121 respondents or assess its potential impact on the results. Nevertheless, such an imbalance may introduce biased perspectives that disproportionately reflect the practices and priorities of overrepresented regions, potentially limiting the global applicability of the findings and leading to recommendations that may be less relevant or feasible in underrepresented areas, where healthcare systems, resources, and patient needs can differ significantly (Pardhan et al. 2025).

The characteristics of the respondents were limited to two questions regarding professional specialty and working environment. It is possible that the respondents' age, gender, and years of experience in managing the edentulous maxilla could have an impact on the results as well. In the absence of this information, this could therefore also be a limitation to be considered in the interpretation of the results.

This study employed a focused rather than comprehensive survey, with 22 clinically oriented items aimed at capturing key aspects of implant‐supported prostheses for the edentulous maxilla. Although these items reflect current restorative trends and literature, they primarily address implant‐supported fixed prostheses and should be interpreted accordingly within the wider spectrum of clinical scenarios and decision‐making pathways for the rehabilitation of the edentulous maxilla.

With the wider range of prosthodontic options in mind and after review of the survey results, several gaps in the literature were identified concerning the treatment planning and clinical decision‐making for edentulous maxilla rehabilitation. Consequently, four PICO (Population, Intervention, Comparison, Outcomes) questions have been proposed for further investigation:

*Question 1*: In adults with controlled medical health with a fully edentulous maxilla (P), what is the efficacy of different types of full‐arch implant‐assisted maxillary reconstructions (I and C) in terms of prosthetic survival and/or success after ≥ 3 years of follow‐up (O)?

*Question 2*: In patients with an edentulous maxilla receiving a full‐arch IOD (P), do stud attachments (I) compared to other retention systems such as bar, magnetic, or telescopic attachments (C), improve patient satisfaction and other ClinROs (O)?

*Question 3*: In patients with an edentulous maxilla receiving a fixed full‐arch implant‐supported prosthesis (P), does full zirconia (I) compared to other restorative materials like porcelain‐fused‐to‐metal, composite, acrylic, or PEEK (C), reduce prosthetic complications or improve other ClinROs (O)?

*Question 4*: In patients with an edentulous maxilla receiving a fixed full‐arch implant‐supported prosthesis (P), do bone‐level implants (I) compared to tissue‐level implants (C) improve outcomes such as biological complications, prosthetic complications, or other ClinROs (O)?

The goal of the four specific PICO questions is to address existing gaps in the literature regarding efficacy, clinical outcomes, biological and prosthetic complications, and patient satisfaction in patients with an edentulous maxilla receiving full‐arch implant‐supported prostheses.

## 5 Conclusion

This study outlined the single‐round survey process used to gather expert insights to inform a consensus development process suggested by the GCCG initiative to support the establishment of a clinical practice guideline for implant‐supported fixed and removable prostheses of the edentulous maxilla. The survey identified 18 statements from 6 items that reached consensus or strong consensus among respondents. The rest of the survey items did not receive consensus, but several came close and achieved majority agreement, while others demonstrated heterogeneity of divided opinions. Four additional research questions were proposed to address existing gaps in the literature regarding prosthetic options, biological and prosthetic complications, clinical outcomes, and patient satisfaction.

## Author Contributions

**Guo‐Hao Lin:** methodology, validation, formal analysis, investigation, data curation, writing – original draft. **Franz J. Strauss:** methodology, software, validation, formal analysis, investigation, visualization, data curation, writing – original draft. **Giulia Brunello:** methodology, software, validation, formal analysis, investigation, visualization, data curation, writing – original draft. **Charlotte Stilwell:** conceptualization, data curation, writing – review and editing, supervision. **Ronald E. Jung:** conceptualization, resources, writing – review and editing, project administration, supervision. **Ina Kopp:** conceptualization, methodology, visualization, data curation, writing – review and editing, supervision. **Frank Schwarz:** conceptualization, resources, writing – review and editing, project administration, supervision. **Hom‐Lay Wang:** conceptualization, resources, writing – review and editing, project administration, supervision.

## Ethics Statement

The protocol was notified to and approved by the Ethical Committee of the University of Düsseldorf (Protocol no. 2024‐2973).

## Conflicts of Interest

The authors declare no conflicts of interest.

## Supporting information


**Figure S1:** In future studies on maxillary full‐arch rehabilitation with dental implants, how relevant do you consider the following patient‐reported outcome measures (PROMs)?

**Figure S2:** In future studies on maxillary full‐arch rehabilitation with dental implants, how relevant do you consider the following clinician‐reported outcomes (ClinROs)?

**Appendix S1:** clr70027‐sup‐0003‐AppendixS1.pdf.

**Appendix S2:** clr70027‐sup‐0004‐AppendixS2.pdf.

## Data Availability

The data that support the findings of this study are available from the corresponding author upon reasonable request.
