# Supplementary material for: 1st Global Consensus for Clinical Guidelines for the Rehabilitation of the Edentulous Maxilla: Single‐Round Survey on Implant‐Supported Fixed and Removable Prostheses
Source: Clin Oral Implants Res. 2026 Feb 24;37(Suppl 30):S121–34. doi: 10.1111/clr.70027 (PMC12930127; doi:10.1111/clr.70027)

Supplemental Figure 1. In future studies on maxillary full-arch rehabilitation with dental implants, how relevant do you consider the following patient-reported outcome measures (PROMs)?

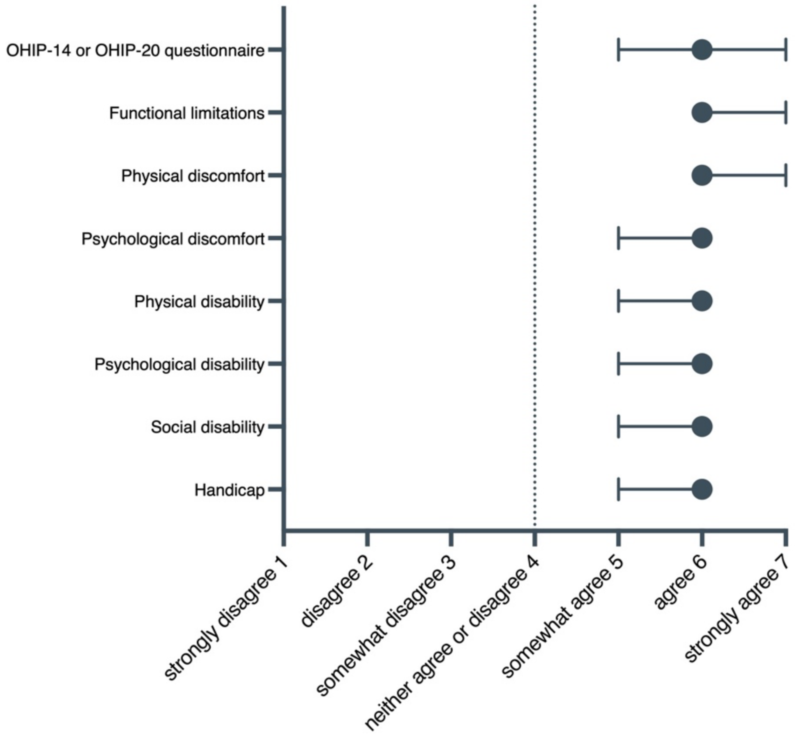

Supplement: Supplementary file 1 — Figure S1: In future studies on maxillary full‐arch rehabilitation with dental implants, how relevant do you consider the following patient‐reported outcome measures (PROMs)? [file CLR-37-S121-s002.pdf]
