# Supplementary material for: 1st Global Consensus for Clinical Guidelines for the Rehabilitation of the Edentulous Maxilla: Single‐Round Survey on Implant‐Supported Fixed and Removable Prostheses
Source: Clin Oral Implants Res. 2026 Feb 24;37(Suppl 30):S121–34. doi: 10.1111/clr.70027 (PMC12930127; doi:10.1111/clr.70027)
Supplement: Supplementary file 3 — Appendix S1: clr70027‐sup‐0003‐AppendixS1.pdf. [file CLR-37-S121-s003.pdf]

Survey 4 – 1st Global Consensus for Clinical Guidelines 2025

Implant supported fixed, removable and conventional overdentures

* Required

1. If you click on "yes", it means that you consent to participate in the survey, otherwise it ends here. *

   - ◯ Yes
   - ◯ No

2. Professional specialisation *

   Multiple choice possible

   - ☐ Periodontology
   - ☐ Prosthodontics
   - ☐ Oral surgery
   - ☐ Oral and maxillofacial surgery
   - ☐ None (general practitioner)
   - ☐ Other

3. Please specify your professional working environment *

   Multiple choice possible

   - ☐ University
   - ☐ Public hospital
   - ☐ Private clinic
   - ☐ Other

4. For a fixed, complete implant supported maxillary prosthesis, where and when do you utilize "multi-unit abutments"? *

   - ◯ On all implants
   - ◯ Never
   - ◯ Only on significantly angled implants
   - ◯ Only on anterior implants
   - ◯ Other

5. For a fixed, complete implant supported maxillary prosthesis, what is your preferred implant type? *

   - ◯ Bone level
   - ◯ Tissue level
   - ◯ Ambivalent
   - ◯ Other

6. For an asymptomatic patient with a fixed, complete implant supported maxillary prosthesis, at what interval do you remove the definitive prosthesis for evaluation, hygiene, or screw replacement? *

- ◯ Every visit
- ◯ Yearly
- ◯ Every 1-3 years
- ◯ Every 3-5 years
- ◯ Only when signs or symptoms require it
- ◯ Never
- ◯ Other

7. For a <u>fixed</u>, complete implant supported prosthesis, what is your preferred material / design in the maxilla? (assuming an opposing fixed, complete implant supported prosthesis). *

- ◯ Full zirconia, with no titanium bases or abutments
- ◯ Full zirconia, with titanium bases or abutments
- ◯ Zirconia superstructure with a custom titanium framework
- ◯ Ceramo-metal (PFM)
- ◯ Acrylic over metal framework
- ◯ PEEK (poly-ether-ether-ketone) based prosthesis
- ◯ Other

8. For a <u>fixed</u>, complete implant supported prosthesis, what is your preferred retention method in the maxilla? (assuming an opposing fixed, complete implant supported prosthesis). *

- ◯ Cemented intra-orally on abutments
- ◯ Screw retained
- ◯ Other

9. Do you commonly utilize a removeable, implant retained (i.e., locators or bar), full arch prosthesis for the treatment of the edentulous maxilla? *

Choose all that apply

- ☐ Yes, wherever possible. It is my preferred treatment for the edentulous maxilla
- ☐ Only when patient finances dictate
- ☐ When alveolar volume in the anterior is insufficient for adequate support of the lip
- ☐ Almost never
- ☐ Never

10. For a fixed, complete implant supported prosthesis, do you routinely re-torque the prosthetic screws at a follow-up appointment after placement? *

- Yes, within 1 month of delivery of the prosthesis
- Yes, within 1 year of delivery of the prosthesis
- Almost never
- Never

11. For a fixed, complete implant supported zirconia prosthesis, do you routinely use titanium abutments or bases in the prosthesis? *

- Always
- Sometimes
- Never
- Not applicable, I don’t use zirconia prostheses

12. If you commonly utilize a single piece, full arch implant fixed prosthesis in the edentulous maxilla, do you utilize a milled titanium framework? *

- Always
- Sometimes
- Never
- Not applicable, I don’t use zirconia prostheses

13. In order to fabricate the master model for the fabrication of the definitive prosthesis (with 4 or more implants), what is your preferred impression or scan method? *

- Closed tray impression copings
- Open tray impression copings (unsplinted)
- Open tray impression copings (splinted)
- Intraoral scan
- Photogrametery with intraoral scan for soft tissues
- Other

14. For multiple implants planned to be part of the same prosthesis, what is the maximum number of implants you would use an intra-oral scanner (not photogrammetry) on to fabricate the master cast? *

- 2 (single quadrant)
- 3 (single quadrant)
- 4 (complete arch)
- 5 (complete arch)
- Any number (complete arch)
- I do not use intra oral scanners

15. For a fixed, complete implant supported maxillary prosthesis, assuming sufficient bone exists or can be augmented, how far distally do you want the distal most implant? *

- ◯ Second premolar
- ◯ First molar
- ◯ Second molar
- ◯ Other

16. For a fixed, complete implant supported prosthesis, what anatomical landmark(s) do you use to set the teeth positions? *

Choose all that apply

- ☐ Facial midline and upper lip
- ☐ Retromolar pad
- ☐ Eyes
- ☐ Lips
- ☐ Nose
- ☐ Other

17. For a fixed, complete implant supported maxillary prosthesis, what is your commonly preferred timing for delivery of the provisional? *

- ◯ Same day as implant placement
- ◯ Day after implant placement
- ◯ Within the first week
- ◯ After implant osseointegration
- ◯ Other

18. Do you commonly use a conventional removable denture and convert it to fixed for the provisional? *

- ◯ Yes, always
- ◯ Sometimes
- ◯ Never

19. For a fixed, complete implant supported prosthesis, do you commonly have it fabricated as a single prosthesis, or do you use segmentation? *

- ◯ Always as a complete arch, 1 piece
- ◯ Mostly as a complete arch, 1 piece
- ◯ Occasionally as a complete arch, 1 piece
- ◯ Never. I always segment the prosthesis

20. For a fixed, complete implant supported prosthesis, what is your preferred home hygiene instrument / regimen? *

Choose all that are commonly preferred

- [ ] Manual toothbrush
- [ ] Electric toothbrush
- [ ] "Super floss"
- [ ] Water flosser (i.e., "Waterpik")
- [ ] Proxy brushes
- [ ] Other

21. Do you routinely use an angled screw system as part of the definitive, complete arch, maxillary prosthesis? *

- ◯ Yes, routinely
- ◯ Sometimes
- ◯ Rarely
- ◯ Never

22. For a fixed, complete implant supported prosthesis, what do you believe (or have seen) to be the most likely or frequent complication? *

- ◯ Fracture of veneering porcelain
- ◯ Framework fracture
- ◯ Loose screws
- ◯ Broken screws
- ◯ Debonding of titanium bases or abutments

23. For a fixed, complete implant supported maxillary prosthesis, what is the smallest implant diameter you would plan to support it in an otherwise "normal" or healthy occlusal scenario? Assuming sufficient number of implants. *

- ◯ 2mm
- ◯ 2.9 or 3mm
- ◯ 3.5mm
- ◯ 4mm
- ◯ Other

24. In future studies on maxillary full-arch rehabilitation with dental implants, how relevant do you consider the following patient reported outcome measures (PROMs)? *

Per each answer, please indicate your level of agreement with a score from "strongly disagree" to "strongly agree" ***Make sure to slide the table all the way to the right to see all score levels, including "strongly agree."***

	strongly disagree	disagree	somewhat disagree	neither agree or disagree	somewhat agree	agree	strongly agree
OHIP-14 or OHIP-20 questionnaire	◯	◯	◯	◯	◯	◯	◯
Functional limitations	◯	◯	◯	◯	◯	◯	◯
Physical discomfort	◯	◯	◯	◯	◯	◯	◯
Psychological discomfort	◯	◯	◯	◯	◯	◯	◯
Physical disability	◯	◯	◯	◯	◯	◯	◯
Psychological disability	◯	◯	◯	◯	◯	◯	◯
Social disability	◯	◯	◯	◯	◯	◯	◯
Handicap	◯	◯	◯	◯	◯	◯	◯

25. In future studies on maxillary full-arch rehabilitation with dental implants, how relevant do you consider the following clinician reported outcome measures (CROMs)? *

Per each answer, please indicate your level of agreement with a score from "strongly disagree" to "strongly agree" ***Make sure to slide the table all the way to the right to see all score levels, including "strongly agree."***

	strongly disagree	disagree	somewhat disagree	neither agree or disagree	somewhat agree	agree	strongly agree
Implant survival	◯	◯	◯	◯	◯	◯	◯
Implant success	◯	◯	◯	◯	◯	◯	◯
Prosthesis survival	◯	◯	◯	◯	◯	◯	◯
Peri-implant bone level	◯	◯	◯	◯	◯	◯	◯
Biological complications	◯	◯	◯	◯	◯	◯	◯
Prosthetic complications	◯	◯	◯	◯	◯	◯	◯
Pocket depth	◯	◯	◯	◯	◯	◯	◯
Plaque index	◯	◯	◯	◯	◯	◯	◯
Gingival index	◯	◯	◯	◯	◯	◯	◯
Bleeding on Probing	◯	◯	◯	◯	◯	◯	◯

This content is neither created nor endorsed by Microsoft. The data you submit will be sent to the form owner.

Microsoft Forms
