# Supplementary material for: 1st Global Consensus for Clinical Guidelines for the Rehabilitation of the Edentulous Maxilla: Single‐Round Survey on Implant‐Supported Fixed and Removable Prostheses
Source: Clin Oral Implants Res. 2026 Feb 24;37(Suppl 30):S121–34. doi: 10.1111/clr.70027 (PMC12930127; doi:10.1111/clr.70027)
Supplement: Supplementary file 4 — Appendix S2: clr70027‐sup‐0004‐AppendixS2.pdf. [file CLR-37-S121-s004.pdf]

Appendix 2: A summary of the results for all items.

Consent and Professional Background			
a) Declaration of consent (item 1)	Out of 230 experts contacted, 123 accessed the survey. Of these, 121 provided consent and completed the questionnaire, resulting in a response rate of 52.6%.		
b) Professional specialization and working environment (items 2-3)	The majority of respondents (71.9%) held a specialization degree, with 21.5% having multiple specialties. Prosthodontics was the most common specialty (67.8%), followed by Periodontology (24.8%), Oral Surgery (19.8%), and Oral & Maxillofacial Surgery (4.1%). Most respondents worked in universities (70.2%) and private clinics (66.9%), with about half (52.1%) practicing in a single setting.		
	Strong Consensus	***Consensus***	***No Consensus but with Majority Agreement***
**Patient Selection**			
c) Use of IODs (item 9)	*NA*	*NA*	*Only when alveolar volume was insufficient for lip support (64.5%)*
**Diagnostic Tools**			
d) Anatomical landmark for tooth positioning(s) (item 16)	*NA*	*Facial midline and upper lip (94.2%)*	*Lips (67.8%), eyes (66.9%)*
**Treatment Planning**			
e) Preferred implant type (item 5)	*NA*	*NA*	*Preferred bone-level implants (66.1%)*
f) Smallest implant diameter (item 23)	*NA*	*3.5 mm (81.0%)*	*NA*
g) Most distal implant site for maxillary fixed prostheses (item 15)	*NA*	*First molar position (81.8%)*	*NA*
h) Preferred retention method (item 8)	*NA*	*Screw-retained prostheses (93.4%)*	*NA*
i) Utilization of an angled screw channel systems (item 21)	*NA*	*NA*	*Sometimes (59.5%)*
j) Timing for delivery of the provisional prosthesis (item 17)	*NA*	*NA*	*NA*
k) Utilization of a conventional removable denture for conversion (item 18)	*NA*	*NA*	*Sometimes (61.2%)*
l) Fabrication of a single prosthesis or segmented prostheses (item 19)	*NA*	*NA*	*Mostly (54.5%)*
m) Preferred prosthesis material/design in the maxilla (item 7)	*NA*	*NA*	*NA*

n) Utilization of milled titanium framework (item 12)	*NA*	*NA*	*Sometimes (60.3%)*
o) Utilization of titanium abutments or bases in the prostheses (item 11)	*NA*	*NA*	*Always (66.1%)*
p) Utilization of multi-unit abutments (item 4)	*NA*	*NA*	*Used on all implants (62.0%)*
**Treatment Procedures**			
q) Impression or scan method (item 13)	*NA*	*NA*	*NA*
r) Maximum number of implants when using intraoral scanner (item 14)	*NA*	*NA*	*NA*
**Maintenance Care**			
s) Home hygiene instruments/regimens (item 20)	*NA*	*NA*	*Super floss (72.7%), proxy brushes (72.7%), electric toothbrushes (61.2%), water flossers (58.7%)*
t) Interval of removing the definitive prosthesis for evaluation, hygiene, or screw replacement (item 6)	*NA*	*NA*	*NA*
u) Frequency of re-torquing the prosthetic screws (item 10)	*NA*	*NA*	*NA*
v) Most encountered or frequent complication (item 22)	*NA*	*NA*	*Fracture of veneering porcelain (58.7%)*
**Fundamental Outcomes to Be Included in Future Studies**			
w) Relevant patient-reported outcome measures (PROMs) (item 24)	*Functional limitations (95.3%)*	*OHIP-14 or OHIP-20 questionnaires (82.6%), physical discomfort (91.7%), psychological discomfort (85.9%), physical disability (81.8%), psychological disability (81.0%), social disability (86.8%), handicap (79.4%)*	*NA*
x) Relevant clinician-reported outcomes (ClinROs) (item 25)	*Prosthesis survival (96.7%), peri-implant bone level (97.5%), biological complications (97.5%),*	*Implant survival (92.6%), implant success (94.2%), pocket depth (85.1%), plaque index (90.9%), gingival index (86.7%),*	*NA*

	prosthetic complications (96.8%)	*bleeding on probing (88.4%)*	
***NA:** Not available, indicating no strong consensus, consensus or majority agreement reached in this category*			
